# Supplementary material for: Causal Relationship between Aging and Anorexia Nervosa: A White-Matter-Microstructure-Mediated Mendelian Randomization Analysis
Source: Biomedicines. 2024 Aug 16;12(8):1874. doi: 10.3390/biomedicines12081874 (PMC11351342; doi:10.3390/biomedicines12081874)
Supplement: Supplementary file 1 [file biomedicines-12-01874-s001.zip › Supplementary Figure/Figure S8/Leaveoneout plot of MR analysis for TL to ubm-b-1891.pdf]

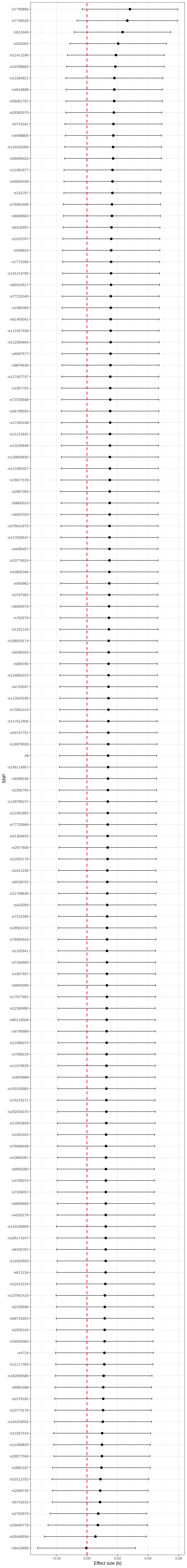
rs7790856
rs7705526
rs611646
rs932002
rs11412296
rs10768683
rs11584821
rs4616688
rs56061761
rs28363070
rs4731541
rs4498805
rs142426306
rs59409453
rs11991877
rs45604339
rs131797
rs78491606
rs6669563
rs8102497
rs1023767
rs939916
rs7772289
rs141214782
rs80324517
rs77231040
rs1985369
rs61405042
rs111527438
rs112394943
rs6587577
rs9878436
rs117407747
rs1907702
rs73730598
rs56799554
rs17445108
rs11212631
rs13230646
rs139669835
rs111950327
rs79977579
rs2967355
rs9600019
rs6007020
rs376641875
rs117630647
rs4695407
rs10774624
rs10805346
rs450962
rs3767952
rs6584579
rs762679
rs1291143
rs188918174
rs6590343
rs869785
rs116863223
rs4743037
rs113525195
rs73581419
rs117512405
rs55747751
rs16978028
All
rs145114957
rs9398196
rs2282764
rs139795227
rs12451892
rs77732866
rs41304832
rs2977608
rs12932179
rs1611236
rs6536702
rs11769630
rs429358
rs7221585
rs28502153
rs76065543
rs1332941
rs7164950
rs1957937
rs9940099
rs17677991
rs12369950
rs80116508
rs6790988
rs11085072
rs7099229
rs11579626
rs3093888
rs150150565
rs76219171
rs202034370
rs17803849
rs1003322
rs76666449
rs10845387
rs9955360
rs3785074
rs7209057
rs6659669
rs4530278
rs143190905
rs185174247
rs8105767
rs12925933
rs871134
rs12412214
rs137901416
rs2230590
rs66731853
rs2555104
rs34550383
rs4724
rs11117354
rs182059586
rs6881568
rs2276182
rs10773176
rs144204502
rs11557154
rs11699829
rs28577594
rs3891167
rs10112752
rs2056726
rs5742915
rs2763979
rs35640778
rs35446936
rs9419958
SNP
−0.02
0.00
0.02
0.04
0.06
Effect size (b)
